# Genetic Characterization and Evolution of Porcine Deltacoronavirus Isolated in the Republic of Korea in 2022

**DOI:** 10.3390/pathogens12050686

**Published:** 2023-05-07

**Authors:** Hye-Ryung Kim, Jonghyun Park, Kyoung-Ki Lee, Hye-Young Jeoung, Young S. Lyoo, Seung-Chun Park, Choi-Kyu Park

**Affiliations:** 1 College of Veterinary Medicine & Animal Disease Intervention Center, Kyungpook National University, Daegu 41566, Republic of Korea; gpfuddl25@knu.ac.kr (H.-R.K.); parkjh@knu.ac.kr (J.P.); parksch@knu.ac.kr (S.-C.P.); 2 DIVA Bio Incorporation, Daegu 41519, Republic of Korea; 3 Animal and Plant Quarantine Agency, Gimcheon 39660, Republic of Korea; naturelkk@korea.kr (K.-K.L.); jhy98@korea.kr (H.-Y.J.); 4 College of Veterinary Medicine, Konkuk University, Seoul 05029, Republic of Korea; lyoo@konkuk.ac.kr; 5 Laboratory of Veterinary Pharmacokinetics and Pharmacodynamics, College of Veterinary Medicine and Cardiovascular Research Institute, Kyungpook National University, Daegu 41566, Republic of Korea

**Keywords:** coronavirus, porcine deltacoronavirus, evolution, phylogenetic analysis

## Abstract

Porcine deltacoronavirus (PDCoV) is an emerging coronavirus that causes diarrhea in nursing piglets. Since its first outbreak in the United States in 2014, this novel porcine coronavirus has been detected worldwide, including in Korea. However, no PDCoV case has been reported since the last report in 2016 in Korea. In June 2022, the Korean PDCoV strain KPDCoV-2201 was detected on a farm where sows and piglets had black tarry and watery diarrhea, respectively. We isolated the KPDCoV-2201 strain from the intestinal samples of piglets and sequenced the viral genome. Genetically, the full-length genome and spike gene of KPDCoV-2201 shared 96.9–99.2% and 95.8–98.8% nucleotide identity with other global PDCoV strains, respectively. Phylogenetic analysis suggested that KPDCoV-2201 belongs to G1b. Notably, the molecular evolutionary analysis indicated that KPDCoV-2201 evolved from a clade different from that of previously reported Korean PDCoV strains and is closely related to the emergent Peruvian and Taiwanese PDCoV strains. Furthermore, KPDCoV-2201 had one unique and two Taiwanese strain-like amino acid substitutions in the receptor-binding domain of the S1 region. Our findings suggest the possibility of transboundary transmission of the virus and expand our knowledge about the genetic diversity and evolution of PDCoV in Korea.

## 1. Introduction

Porcine deltacoronavirus (PDCoV) is an emerging enteropathogenic coronavirus that causes typical clinical symptoms, including severe diarrhea, vomiting, dehydration, and mortality in seronegative neonatal piglets [1,2,3,4,5]. PDCoV is an enveloped, single-stranded, positive-sense RNA virus in the genus Deltacoronavirus, family Coronaviridae, of the order Nidovirales, with a genome of approximately 25.4 kb in length [4,6]. The genome arrangement of PDCoV is as follows: 5′-untranslated region (UTR), open reading frame 1a/1b (ORF1a/1b), spike (S), envelope (E), membrane (M), nonstructural protein 6 (NS6), nucleocapsid (N), NS7, NS7a, and 3′-UTR [6]. PDCoV was first detected in 2012 in pig rectal swabs collected from Hong Kong as a part of molecular surveillance studies to investigate the diversity of coronaviruses in mammals and birds [6]. In early 2014, an outbreak of PDCoV-associated diarrhea broke out in pig farms in Ohio, United States, which led to the identification of the first clinical case of PDCoV infection in pigs [2,4,7,8]. Since then, PDCoV has been detected worldwide, including in Korea, Japan, China, Thailand, Vietnam, Laos, Peru, and Mexico [3,9,10,11,12,13,14,15,16,17,18,19,20,21,22,23]. According to previous phylogenetic analyses, PDCoV can be divided into three or four groups based on their full-length genome sequences [15,24]. In Korea, PDCoV (KNU14-04 strain; GenBank accession no. KM820765) was first identified in April 2014 from the fecal samples of pigs with diarrhea [17]. Subsequent studies have shown that all Korean PDCoV strains identified during 2014–2016 have a high sequence identity with strains reported in the United States [10,18,21]. However, since 2016, there have been no reports of PDCoV outbreaks in Korea. In June 2022, we detected PDCoV RNAs from diarrheal pigs in a Korean domestic pig farm, successfully isolated the virus (designated KPDCoV-2201), and analyzed its full-length genome sequences to elucidate the genetic characteristics and evolution of KPDCoV-2201 currently detected in Korea.

## 2. Materials and Methods

### 2.1. Sample Collection and RNA Extraction

In June 2022, eight fecal and one intestinal tissue samples were submitted to the Animal Disease Intervention Center of Kyungpook National University from a pig farm located in Gyeongsangbuk-do. Each sample was separately suspended in phosphate-buffered saline, homogenized, and centrifuged. The supernatant of each sample was used to extract total DNA/RNA using Patho Gene-spin™ DNA/RNA Extraction Kit (iNtRON Biotechnology, Sungnam, Korea).

### 2.2. Detection of Enteric Pathogens

To detect swine enteric pathogens, real-time reverse transcription polymerase chain reaction (qRT–PCR) was performed for 10 viral pathogens [PDCoV, porcine epidemic diarrhea virus (PEDV), transmissible gastroenteritis virus (TGEV), porcine enteric alphacoronavirus (PEAV), swine acute diarrhea syndrome coronavirus (SADS-CoV), porcine torovirus (PToV), porcine sapelovirus (PSV), group A rotavirus (RVA), group B rotavirus (RVB), group C rotavirus (RVC)] using a commercial one-step qRT–PCR kit (THUNDERBIRD™ Probe One-step qRT–PCR kit, TOYOBO, Osaka, Japan). The previously reported 10 qRT-PCR assays were performed using the target gene-specific primer and probe sets of each viral pathogen, as described in their studies [7,25,26,27,28,29,30]. The reaction composition and conditions were performed according to the manufacturer’s instructions.

### 2.3. Isolation and Genome Sequencing of PDCoV

Sequence analysis of the viral genome was performed on seven samples with a cycle threshold (Ct) of ≤25 among the nine PDCoV-positive samples. The spike (S) protein genes were sequenced by RT-PCR using PDCoV-specific primer sets (Table S1). We attempted to isolate PDCoV from the PDCoV-positive intestinal samples using swine testis (ST) cells (ATCC CRL-1746), as described previously [31]. We designed 11 pairs of primers for full-length genome sequencing of KPDCoV-2201 based on the conserved regions of the 127 complete genome sequences available in GenBank (Table S1). The synthesis of each cDNA fragment, PCR amplification, purification of the amplified PCR products, and determination of the sequences of the 5′- and 3′-termini of the viral genome were performed as previously described by Park et al. 2022 [32]. The sequences of each product were analyzed using the Sanger’s method by a commercial company (BIONICS, Daejeon, Republic of Korea). One complete genome sequence (strain KPDCoV-2201) was deposited to GenBank (accession numbers OQ718500).

### 2.4. Phylogenetic and Molecular Evolutionary Analyses

To determine the genetic relationship between KPDCoV-2201 and other strains, nucleotide (nt) and amino acid (aa) sequences were aligned using MAFFT [33], which is available in Geneious Prime (https://www.geneious.com, accessed on 21 July 2022), together with 127 other full-length PDCoV genome sequences and 273 complete spike gene sequences available in GenBank. Phylogenetic trees were constructed using the RAxML method available in Geneious Prime (https://www.geneious.com, accessed on 21 July 2022) based on the general time-reversible (GTR) nucleotide substitution with a gamma distribution model [34]. These trees were subjected to bootstrap analysis with 1000 replicates to determine the percentage reliability values for each internal node of the tree [35]. All phylogenetic trees were visualized using the iTOL phylogenetic tree viewer [36]. To estimate the temporal distributions of KPDCoV-2201 and the phylogenetic relationships in South Korea, we constructed a time-scaled phylogenetic tree of G1b PDCoV strains using BEAST v.1.10.4 [37] with a GTR + gamma (GTR + γ) substitution model. Time-scaled maximum clade credibility tree was constructed using TreeAnnotator v.1.10.4 (https://beast.community/treeannotator, accessed on 22 July 2022) in BEAST and was visualized using FigTree v.1.4.3 (http://tree.bio.ed.ac.uk/software/figtree/, accessed on 22 September 2022).

## 3. Results

### 3.1. Detection and Isolation of KPDCoV-2201

**Figure 1:**
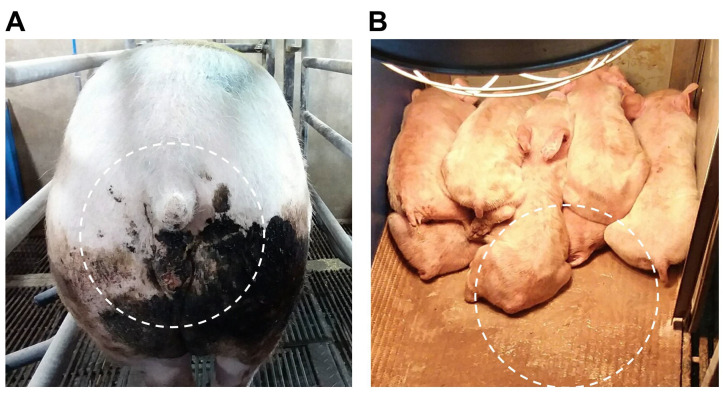
PDCoV affected pigs on the farm of isolate KPDCoV-2201 strain. (**A**) Black tarry diarrhea in sows; (**B**) watery diarrhea in piglets.

In June 2022, pigs of all ages on a farm located in Gyeongsangbuk-do exhibited the clinical symptoms of watery diarrhea. Approximately 10% of the sows presented with diarrhea symptoms, whereas 90% of the piglets showed diarrhea symptoms with a mortality rate of 40%, lower than the typical mortality rate of PEDV infection (Figure 1). Etiological diagnostic results showed that all nine samples collected from the farm were positive for PDCoV but negative for the remaining nine viral antigens (Table 1). Subsequently, seven PDCoV-positive samples with high viral titers (Ct value of <25) were selected for the sequencing of S gene. The results of the sequencing analysis showed that all S genes from all seven samples shared 100% homology at the nucleotide level. Further, we selected the intestinal sample with the highest viral load and inoculated it into ST cells for virus isolation. After one passage of the cell culture, we observed a remarkable cytopathic effect in the infected cells, indicating that the PDCoV Korean isolate was successfully isolated in this study (Figure S1).

### 3.2. Full-Length Genomic Characterization of KPDCoV-2201

The full-length genome sequence of KPDCoV-2201 was analyzed to determine the genetic characteristics of PDCoV, which was recently detected in Korea. The genome of KPDCoV-2201 is 25,422 nt in length, excluding the 3′-poly-(A) tail. It is arranged as follows: 5′-UTR comprising 539 nt, *ORF1a* and *ORF1b* encoding a replicase of 18,803 nt, S gene comprising 3483 nt, E gene comprising 252 nt, M gene comprising 654 nt, NS6 gene comprising 285 nt, N gene comprising 1326 nt, NS7 gene comprising 603 nt, and 3′-UTR comprising 392 nt. The full-length genome of KPDCoV-2201 was submitted to the NCBI GenBank database (GenBank accession no. OQ718500). A comparison of the nucleotide identity of the full-length genome of KPDCoV-2201 showed 96.9–99.2% identity with global PDCoV strains, with 98.7–99.2% identity with Korean PDCoV strains. When the genome of Korean PDCoV strains and that of KPDCoV-2201 were compared, it was found that the M gene was the most conserved, with 99.7–99.8% nucleotide homology. In contrast, the NS7 gene was the most mutated, with 97.5–98.8% nucleotide homology. Interestingly, the E and M genes of KPDCoV-2201 were more similar to those of the Peruvian PDCoV strain (GenBank accession no. MT227371) than to those of other Korean PDCoV strains (Table 2). According to a previously reported structural analysis, the S protein PDCoV contains the S1-NTD (N-terminal domain of S1), S1-CTD (C-terminal domain of S1), CH-N (N-terminal central helix), CH-C (C-terminal central helix), FP (fusion peptide), HR-N (N-terminal heptad repeat), and HR-C (C-terminal heptad repeat), and the PDCoV receptor binding domain (RBD) is located at the C-terminal of the S1 subunit (S1-CTD) of the S protein [38]. Based on the S gene of KPDCoV-2201, the aa sequences of two PDCoV strains, which are closely related to KPDCoV-2201 according to phylogenetic analysis, were aligned and compared with those of the previously reported eight Korean PDCoV strains (Figure S2A). The alignment results revealed that KPDCoV-2201 has 18–31 aa substitutions with Korean PDCoV strains, of which 16 aa substitutions (K96R, V277L, V326I, T387S, R342K, E/K485G, K534N, I550L, A624T, A630T, L632M, N639S, R/Q642K, A706T, K815T, and E854D) were not identical to those of all Korean PDCoV sequences, and 2 aa substitutions (I190V and D284N) were identical to those of only one strain each. KPDCoV-2201 had three aa differences from the previously reported eight Korean PDCoV strains in the S1-CTD region, but two of three were identical to those of the recently emerged PDCoV in the Taiwan strain (GenBank accession no. MZ712038) (Figure S2B).

### 3.3. Phylogenetic and Molecular Evolutionary Analyses

**Figure 2:**
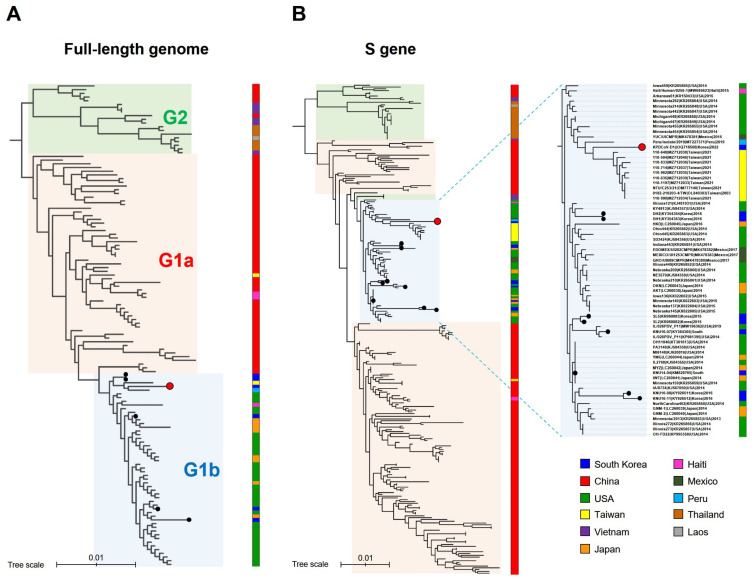
Phylogenetic trees of porcine deltacoronavirus (PDCoV) constructed using 128 global PDCoV full-length genomes (**A**) and 274 S genes (**B**). Red circles indicate the KPDCoV-2201 strain isolated in this study. Black circles indicate the previously reported Korean PDCoV strains. Genogroups (G) of PDCoV are indicated by different background colors of red (G1a), blue (G1b), and green (G2). The countries of the strains are listed on the right side of each strain with the indicated color. The right figure panel is enlarged to depict G1b, including Korean PDCoV strains. Scale bars indicate nt substitutions per site.

For phylogenetic analysis, a RAxML tree was constructed using the genomic sequence of the KPDCoV-2201 strain along with previously reported 127 complete genomic sequences or 273 spike gene sequences, respectively (Figure 2). According to a previous report [15], PDCoV strains were classified into two genogroups (G)s, G1 and G2. G1 is divided into G1a (China) and G1b (United States). G2 includes PDCoV originating from Southeast Asia. Based on phylogenetic analysis of the full-length genome, the PDCoV-2201 strain was classified as G1b clustered with the US-like PDCoVs from the United States, Japan, Taiwan, Peru, Haiti, and the previously reported Korean strains (Figure 2A). Moreover, in the phylogenetic tree constructed using the S gene, KPDCoV-2201 was more closely related to PDCoV strains from Taiwan and Peru and was not clustered with other Korean PDCoV strains (Figure 2B).

**Figure 3:**
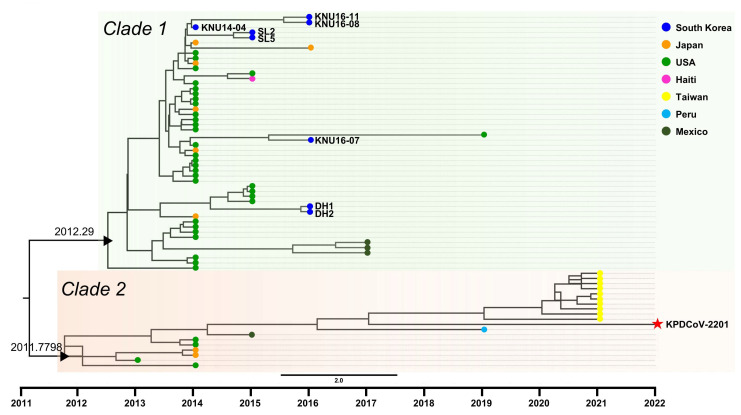
Time-scaled phylogenetic tree of G1b PDCoV strains constructed using BEAST. G1b PDCoV strains could be clustered into two clades based on the divergence of tMRCA. The black triangles represent the tMRCA of each clade. The value of each tMRCA is indicated on the node. Evolutionary clades of G1b PDCoV strains are indicated by different background colors of green (clade 1) and red (clade 2). The countries of the strains are presented on the tips of each strain with the displayed color. A red star indicates the KPDCoV-2201 strain isolated in this study.

To elucidate the evolutionary characteristics of Korean PDCoV strains, times to the most recent common ancestor (tMRCA) were analyzed using the S gene of strains belonging to the G1b group. The tMRCA of G1b was estimated as 2010.856, with a 95% highest posterior density (HPD) ranging from 2008.49 to 2012.77, which diverged to clades 1 and 2. These clades individually had tMRCA between 2011 and 2012. The Korean PDCoV strains that caused outbreaks from 2014 to 2016 belonged to clade 1, which also included most of the American, Japanese, and Mexican strains, and one Haitian strain. The tMRCA of this clade was 2012.29, with a 95% HPD interval ranging from 2011.18 to 2013.31. However, KPDCoV-2201 belonged to clade 2, which included the American, Japanese, and Mexican strains and the Peruvian and Taiwanese strains, which caused the recent outbreaks, with a tMRCA of 2011.78 (95% HPD, 2010.88–2012.57) (Figure 3).

## 4. Discussion

Recent studies have reported recombinant PDCoV strains in Southeast Asia and highly pathogenic strains in China, suggesting that PDCoV exhibits continuous antigenic changes [14,15,39]. Moreover, a case of PDCoV infection in humans was reported in Haiti [40], highlighting the risk of cross-species transmission and zoonosis. Therefore, it is necessary to conduct further research to expand insights into the PDCoV infection status and its genetic epidemiology for the global distribution of the virus.

To our knowledge, there has been no study on PDCoV in Korea for approximately the past 7 years. In 2022, KPDCoV-2201, a Korean PDCoV strain, was isolated from ST cells, and its full-length genome was sequenced in this study. Phylogenetic analysis based on the 128 full-length genome sequences showed that global PDCoV strains were classified into three distinct genogroups: G1a (China group), G1b (United States group), and G2 (Southeast Asia group) as previously reported [14,15] (Figure 2). All Korean PDCoV strains, including KPDCoV-2201 isolated in this study, were classified into the G1b genogroup, but the KPDCoV-2201 was more closely related to Taiwan PDCoV strains than to previously reported Korean PDCoV strains.

Due to research gaps, it remains unclear whether the origin of KPDCoV-2201 is the result of evolutions within Korean PDCoV strains or whether it was recently introduced as part of the Asian epidemic. Therefore, to further elucidate the evolutionary characteristics of the KPDCoV-2201 strain, tMRCA analysis was conducted using the S gene sequences of strains belonging to the G1b group. As a result, the KPDCoV-2201 was classified as Clade 2 and shared the same tMRCA with recently reported Taiwanese and Peruvian strains, which differed from previously reported Korean PDCoV strains belonging to Clade 1 (Figure 3). It is noteworthy that the occurrences of Clade 2 PDCoV strains increased compared to their minor occurrence in 2014-2016. The increasing occurrence of Clade 2 strains may indeed complicate PDCoV epidemiology and make the prevention and control of the disease more challenging.

A previous study reported that the titers of the recently emerged Taiwanese PDCoV strains were higher than those of the strain detected in 2015, indicating severe symptoms due to single infections with these PDCoV strains [41]. In our study, we confirmed that severe clinical symptoms were observed as a consequence of a single infection with PDCoV without coinfection by the other nine types of enteric viral pathogens in sows and piglets. These results suggested that PDCoV could potentially be the primary causative agent of severe diarrhea in some pig farms. However, diagnosing for PDCoV infection may not have been routinely conducted in diagnostic laboratories since its clinical symptoms are similar to those of other major viral pathogens causing severe enteritis, such as PEDV and TGEV. Therefore, it is essential to diagnose PDCoV in domestic pig populations exhibiting the symptoms of diarrhea, and further research is warranted on the pathogenicity of the current circulating PDCoV.

## 5. Conclusions

We isolated the KPDCoV-2201 strain from a Korean domestic pig farm where sows and piglets had severe watery diarrhea. Genetic and evolutionary analysis of KPDCoV-2201 revealed that the newly identified Korean strain is distinct from previously reported Korean strains but is closely related to non-Korean strains derived from Taiwan and Peru. Our findings suggest the possibility of transboundary transmission of the virus and expand our knowledge about the genetic diversity and evolution of PDCoV in South Korea.

## Figures and Tables

**Table 1:** Details and diagnostic results of clinical samples.

Sample	Age	Results of Real-Time Reverse Transcription PCR (qRT-PCR)									
		PEDV	PEAV	TGEV	PToV	PDCoV	SADS	PSV	GARV	GBRV	GCRV
Feces	4 days	N/A	N/A	N/A	N/A	24.75	N/A	N/A	N/A	N/A	N/A
Feces	4 days	N/A	N/A	N/A	N/A	21.08	N/A	N/A	N/A	N/A	N/A
Feces	4 days	N/A	N/A	N/A	N/A	19.04	N/A	N/A	N/A	N/A	N/A
Feces	4 days	N/A	N/A	N/A	N/A	19.87	N/A	N/A	N/A	N/A	N/A
Feces	4 days	N/A	N/A	N/A	N/A	21.84	N/A	N/A	N/A	N/A	N/A
Feces	4 days	N/A	N/A	N/A	N/A	21.96	N/A	N/A	N/A	N/A	N/A
Feces	Sow	N/A	N/A	N/A	N/A	30.32	N/A	N/A	N/A	N/A	N/A
Feces	Sow	N/A	N/A	N/A	N/A	32.29	N/A	N/A	N/A	N/A	N/A
Intestine	4 days	N/A	N/A	N/A	N/A	14.45	N/A	N/A	N/A	N/A	N/A

Abbreviations: PEDV, porcine epidemic diarrhea virus; PEAV, porcine enteric alphacoronavirus; TGEV, transmissible gastroenteritis virus; PToV, porcine torovirus; PDCoV, porcine deltacoronavirus; SADS, swine acute diarrhea syndrome coronavirus; PSV, porcine sapelovirus; GARV, porcine group A rotavirus; GBRV, porcine group B rotavirus; GCRV, porcine group C rotavirus; N/A, not amplified.

**Table 2:** Comparison of the full-length genome of Korean and global PDCoV strains with that of KPDCoV-2201.

Strain/Year	Genbank Accession Number	Country	Full-Length *	5′-UTR	ORF1a	ORF1b	S	E	M	NS6	N	NS7	3′-UTR
% Identity to KPDCoV-2201													
KNU14-04/2014	KM820765	Korea	99.2	99.3	99.2	99.3	98.8	98.8	99.7	98.9	98.8	98.8	98.0
KNU16-07/2016	KY364365	Korea	99.2	99.3	99.2	99.3	98.7	98.8	99.7	98.9	98.5	98.3	98.2
KNU16-11/2016	KY926512	Korea	98.7	98.5	98.9	98.9	97.9	98.4	99.8	98.6	97.9	97.5	97.4
DH1/2016	KY354363	Korea	99.1	99.1	99.2	99.2	98.6	98.8	99.7	99.6	98.8	98.7	98.2
DH2/2016	KY354364	Korea	99.1	99.1	99.2	99.2	98.6	98.8	99.7	99.6	98.8	98.7	98.2
AH2004/2004	KP757890	China	98.4	99.1	98.4	98.8	97.4	97.6	98.9	98.9	97.7	97.8	97.5
HKU15-155/2010	NC_039208	China	98.6	98.8	98.7	98.9	97.6	98.4	99.1	98.2	98.3	98.2	98.0
Sichuan/2019	MK993519	China	97.7	98.3	97.5	98.2	97.4	98.4	98.5	98.6	97.9	98.0	94.9
IL2768/2014	KJ584355	United States	99.2	99.3	99.3	99.3	98.8	98.8	99.8	99.3	98.6	98.3	98.2
YMG/2014	LC260044	Japan	99.2	^†^	99.3	99.4	98.8	98.8	99.8	98.9	98.8	98.8	98.2
HaNoi6/2015	KX834351	Vietnam	97.8	98.0	96.9	97.8	96.1	98.8	98.9	98.6	98.2	97.7	98.0
S5015L/2015	KU051649	Thailand	96.9	98.5	96.7	97.5	95.8	98.4	98.3	98.2	96.5	96.7	95.9
P1_16_BTL_0115/2016	KX118627	Laos	97.0	98.3	96.7	97.6	96.0	98.4	98.3	98.2	97.8	97.2	95.9
Peru_isolate_2019/2019	MT227371	Peru	99.2	^†^	99.3	99.3	98.6	99.2	100	99.6	98.9	98.8	^†^
NTU_C253_21/2021	OM777140	Taiwan	99.0	^†^	99.1	99.1	98.5	98.8	99.8	98.9	98.8	98.5	98.5

* To avoid errors caused by UTR, the full-length genome identity was evaluated except for the UTR sequence. ^†^ UTR sequence was not fully sequenced.

## Data Availability

Not applicable.

## Supplementary Materials

The following supporting information can be downloaded at: https://www.mdpi.com/article/10.3390/pathogens12050686/s1, Table S1: Primer sets for the complete genome sequencing of PDCoV in this study; Figure S1: KPDCoV-2201 strain causes cytopathic changes in ST cells; Figure S2: Alignment of the amino acid sequences of spike protein in KPDCoV-2201 with Korean PDCoV strains (A). Details of amino acid substitutions of KPDCoV-2201 that differ from KNU14-04 (B).
