# Supplementary material for: Genetic Characterization and Evolution of Porcine Deltacoronavirus Isolated in the Republic of Korea in 2022
Source: Pathogens. 2023 May 7;12(5):686. doi: 10.3390/pathogens12050686 (PMC10222072; doi:10.3390/pathogens12050686)
Supplement: Supplementary file 1 [file pathogens-12-00686-s001.zip › Supplementary Figure S1.pdf]

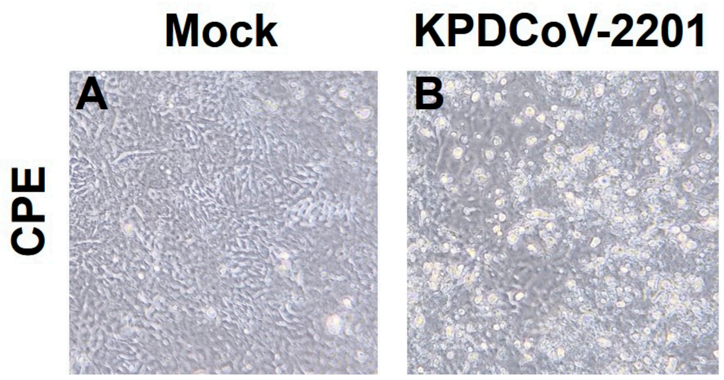

Figure S1. KPDCoV-2201 strain causes cytopathic changes in ST cells. (A) Morphology of Mock-infected ST cells. (B) Morphology of ST cells after 48 h of infection with KPDCoV-2201, showing cell rounding.
