# Supplementary material for: Genetic Characterization and Evolution of Porcine Deltacoronavirus Isolated in the Republic of Korea in 2022
Source: Pathogens. 2023 May 7;12(5):686. doi: 10.3390/pathogens12050686 (PMC10222072; doi:10.3390/pathogens12050686)
Supplement: Supplementary file 1 [file pathogens-12-00686-s001.zip › Supplementary Figure S2.pdf]

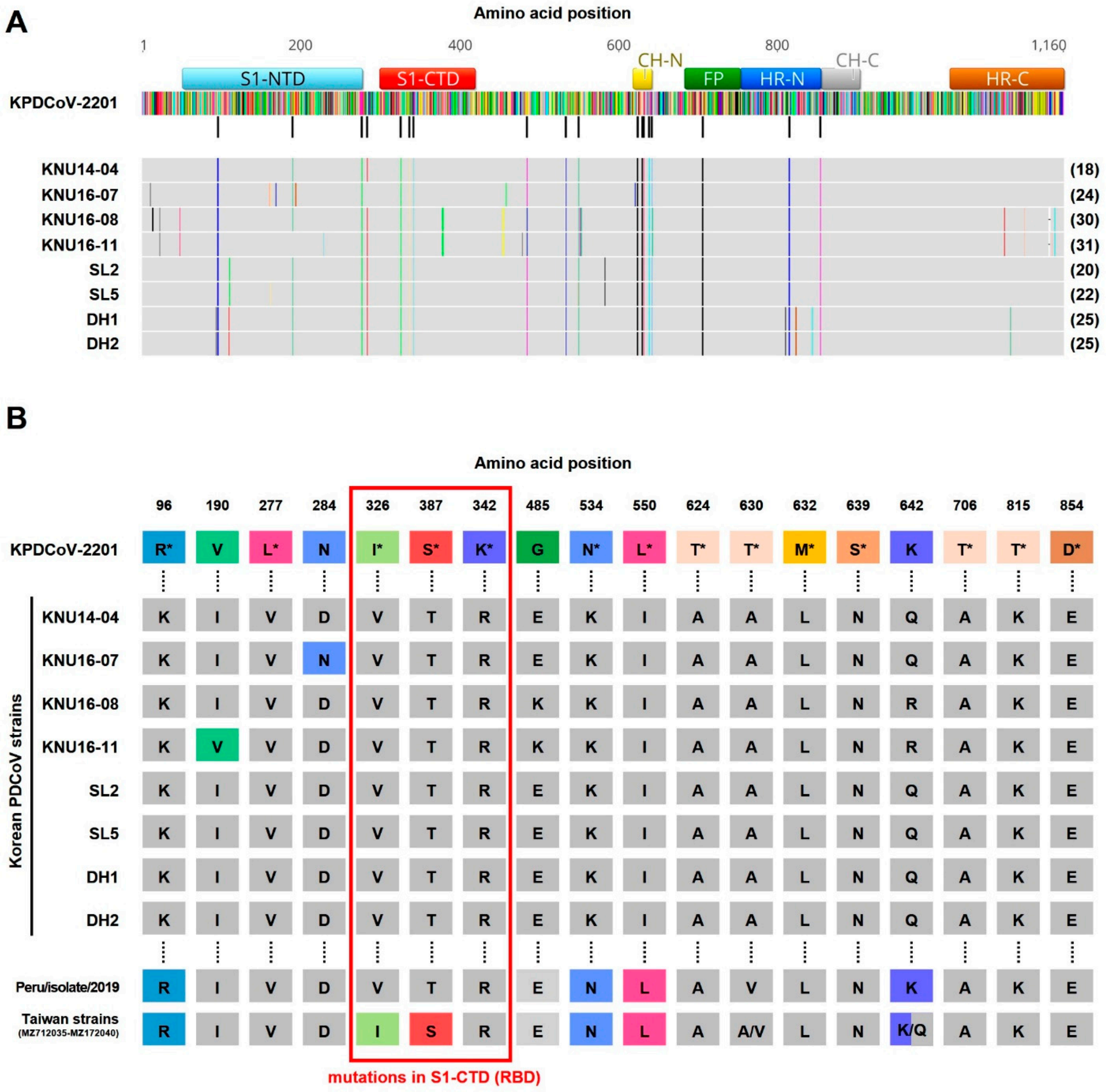

Figure S2. Alignment of the amino acid sequences of spike protein in KPDCoV-2201 with Korean PDCoV strains (A). Details of amino acid substitutions of KPDCoV-2201 that differ from KNU14-04 (B). (A) The top schematic diagram of the PDCoV spike (S) protein ectodomain. S1-NTD, N-terminal domain of S1. S1-CTD, C-terminal domain of S1. RBD receptor binding domain. CH-N N-terminal central helix. CH-C C-terminal central helix, FP fusion peptide, HR-N N-terminal heptad repeat, HR-C C-terminal heptad repeat. Red strain name indicates KPDCoV-

2201 strain in this study. The black bar below the KPDCoV-2201 strain represents the amino acid (aa) substitutions that differ from KNU14-04 (the Korean prototype strain). Grey areas represent identical aa sequences to KPDCoV-2201, and each vertical-colored bars represent aa sequences divergent from KPDCoV-2201. Numbers in parentheses on the right indicate the aa differences compared with KPDCoV-2201. (B) The upper colored amino acids represent 18 amino acid substitutions that are different from KNU14-04 (the Korean prototype strain). Amino acids marked with an asterisk denote substitutions that are unique to the Korean strains. Each aa substitutions were compared with PDCOV strains from Korea, Peru, and Taiwan. At each aa positions, identical amino acids are colored, and different amino acids are colored grey. Red box indicates aa substitutions at S1-CTD (RBD).
