# Supplementary material for: Genetic Characterization and Evolution of Porcine Deltacoronavirus Isolated in the Republic of Korea in 2022
Source: Pathogens. 2023 May 7;12(5):686. doi: 10.3390/pathogens12050686 (PMC10222072; doi:10.3390/pathogens12050686)
Supplement: Supplementary file 1 [file pathogens-12-00686-s001.zip › Supplementary Table S1.pdf]

**Table S1. Primer sets for the complete genome sequencing of PDCoV in this study.**

| Target | Primer | Sequence (5′–3′) | Amplified length |
|---|---|---|---|
| Spike | Spike-1-Fwd | TTGACACCAACCTATTCTCCTTT | 2323 bp |
| | Spike-1-Rev | CATTTTCTCAGCATCAACAACACC | |
| | Spike-2-Fwd | GCAAGTTCAAGCAGAGCAAGTTAT | 1769 bp |
| | Spike-2-Rev | GCAATAATATATTGTCCAGGGATG | |
| Full-length | 1F-Fwd | ACATGGGGACTAAAGATAAAAATTATAGC | 2087 bp |
| | 1F-Rev | CTAAGTACTTCGTCGCTGCTG | |
| | 2F-Fwd | CCATTGATCTACCTGAAGACTATC | 2622bp |
| | 2F-Rev | GCAGACTAACTACAGCTGGTAAC | |
| | 3F-Fwd | GATTATCTGGACATTTTGGATCTTTGG | 2618bp |
| | 3F-Rev | ATGTGAATACCAGATACAGTGCC | |
| | 4F-Fwd | AGACGGTCTCATACTTAACATTTATG | 2554bp |
| | 4F-Rev | CATAACCATATTGAAACCACTGCAT | |
| | 5F-Fwd | GTTCTTCCACTTCTTGTTTATGGCT | 2618bp |
| | 5F-Rev | CACACTGCTTAGTAACAAAGTACAAT | |
| | 6F-Fwd | CCTGTTTCCTTCGCCTTGACT | 2596 bp |
| | 6F-Rev | CCAGAATGCGAGAGACATCTG | |
| | 7F-Fwd | GTTTCCGAGACATTTTGTTTTACCAA | 2557bp |
| | 7F-Rev | GTGTTAGGTTTTATGGCGTGTG | |
| | 8F-Fwd | GCTTTGACCTGGCATGATTGTG | 2549bp |
| | 8F-Rev | GTGTAGGAATGATAATTAGGGATGT | |
| | 9F-Fwd | CAAAGTATTCACTGTGTCAATTGAC | 2597bp |
| | 9F-Rev | GATGGTCGAGTATTCTGTAATGTG | |
| | 10F-Fwd | GAAACCCCTTCTCAACTGATTG | 2540bp |
| | 10F-Rev | CGAATTGAGTCATACCAGTACTT | |
| | 11F-Fwd | CTGCTCCAACCCTTCACCC | 2281bp |
| | 11F-ReV | TTTTTTTTTTGCTCCATCCCCCCTATAAG | |

Abbreviations: PDCoV, porcine deltacoronavirus; F, fragment; Fwd, forward; Rev, reverse; bp, base pair
